# Prevalence of Extended Spectrum β-Lactamase Producers (ESBLs) with antibiotic resistance pattern of Gram negative pathogenic bacteria isolated from door handles in hospitals of Pokhara, Western Nepal

**DOI:** 10.1186/s43141-023-00616-4

**Published:** 2023-11-24

**Authors:** Binita Koirala Sharma, Birendra Prasad Sharma, Anjeela Kunwar, Nirmala Basnet, Padam Darlami Magar, Sajana Adhikari

**Affiliations:** 1 Department of Microbiology, Prithivi Narayan Campus, Tribhuvan University, Pokhara, Nepal; 2 Department of Microbiology, Janapriya Multiple Campus, Tribhuvan University, Pokhara, Nepal

**Keywords:** Contamination, Drug-resistant, Door-Handles, ESBLs, Hospitals

## Abstract

**Background:**

The presence of drug-resistant Gram-negative pathogenic bacteria and Extended Spectrum β-Lactamase Producers (ESBLs) in hospital associated fomites like door handles can serve as vehicles in transmission and may be the key factor in epidemiology of ESBL producing bacterial infection not only in a hospital setting but also in the community. The aim of this study was to determine the prevalence of ESBLs and antibiotic resistance of Gram-Negative pathogenic Bacteria isolated from door-handles in two selected hospitals in Pokhara Metropolitan City, Nepal. The study was conducted in selected hospitals in Pokhara Metropolitan City, Western Nepal**.** A cross-sectional study design was used. The hospitals were selected randomly. A total of 100 swab samples were taken from door-handles. Isolation and identification of bacteria were done using standard microbiological procedures. An antibiotic susceptibility test, screening and confirmation of ESBLs were performed using the Clinical Laboratory Standard Institute’s guidelines.

**Results:**

Out of the 100 swab samples cultured, 96 (96%) showed bacterial growth. A total of one hundred and forty isolates were isolated in this study which were further identified based on cultural, morphological and biochemical characteristics. The study also found that door handles/knobs had higher level of contamination in Outpatient Departments (OPDs), Emergency, Laboratory, General wards and Toilets, in that order as compared to Radiology Room, Staff rooms, Intensive Care Unit and Operation Theatre which were lower. The level of contamination varies depending on the traffic exposure and the environment. The most prevalent Gram-negative bacteria identified was *Escherichia coli* 28.85%, followed by *Klebsiella* spp 21.15%, *Pseudomonas aeruginosa* 15.38%, *Proteus* spp 11.54%, *Enterobacter* spp 9.62%, *Acenetobacter* spp 7.69%, *Citrobacter* spp 5.77%. The most effective drug of choice was Amikacin, Nitrofurantoin, Norfloxacin, Ciprofloxacin, Tetracycline and Imipenem for many Gram-negative isolates. The overall prevalence of ESBLs in this study was 27.14%. Out of total 15 *Escherichia coli* isolated, 11(73.3%), *Klebsiella* spp 9/11 (81.8%); *Pseudomonas* spp 7/8 (87.5%), *Proteus* spp 4/6 (66.6%); *Enterobacter* spp 3/5 (60%), *Acenetobacter* spp 3/4 (75%) and *Citrobacter* spp 1/3 (33.3%) were found to be Extended β-Lactamase Producers (ESBLs).

**Conclusion:**

The isolation of of pathogenic Gram-negative bacteria and ESBLs in hospital environments and subsequent detection of high drug resistance patterns indicates a potentially serious public health challenge that strengthens the need for the effective and routine cleaning of door-handles in hospitals.

## Background

The microorganisms have been frequently isolated from environmental sources that serve as a relay for the bacteria and play major role in its spread of infections between different hosts [1]. Surfaces of the hospitals are often contaminated with microbial flora excreted by patients, visitors and healthcare workers. The contaminated environmental surfaces are not only potential reservoirs for spread of microbial agents inside hospital but also in community. Resistance of pathogenic microorganisms in hospital environment increases the risk of infection among susceptible host [2]. Fomite is one of the major causes of Hospital Acquired Infection that is associated with patient morbidity and mortality [3, 4]. Among the vast range of fomites, door handles may be one of the most common one route for contamination. Door handles contamination with antibiotic resistant bacteria could be a major threat to public health, as the antibiotic resistant determinants could be transferred to other pathogenic bacteria and compromise the treatment of severe bacterial infections and enhancing resistance dissemination [5]. Not much attention is paid in the cleaning of the door handles in hospitals that might lead to increasing and evolve into more pathogenic form of microorganisms.

Inadequate treatment regimens, insufficient patient adherence, unregulated drug distribution and trafficking, as well as antibiotic scarcity and poor quality, can all contribute to antibiotic resistance. Target alteration of the drug, bypassing the drug, the impermeability of the bacteria, biofilm formation, efflux of the drug, mutations and plasmid-mediated transfer of resistance genes are major mechanisms of the antibiotic resistance [5, 6]. Gram negative bacteria may acquire resistance to antibiotics with production of enzymes like beta-lactamase, carbapenemase, and aminoglycoside modifying enzymes, increased expression of the transmembrane efflux pump and alteration in the outer membrane such as porin mutations [7].

Thus, contamination of fomites like door handles with antibiotic resistant bacteria can be a major threat to public health [8]. Members of family Enterobacteriaceae can produce extended spectrum of beta-lactamase which is responsible for the hydrolysis of cephalosporin group antibiotics of the third generation which results in treatment failure [9, 10]. Hospital door handles contaminated with ESBL-producing isolates increase the risk for infection due to ESBL and may be the key factor in the epidemiology of ESBL producing bacterial infection not only in a hospital setting but also in the community. The major cause of emergence of drug resistant microorganisms to antibiotics is the spread of the plasmid encoded Extended Spectrum Beta Lactamase (ESBL) genes that confer resistance to third generation cephalosporins [11]. Treatement options for infections due to ESBL producers have also become increasingly limited [12–14].

Bacterial pathogens that have been isolated from door handles in previous studies included *S. aureus, K. pneumoniae, Escherichia coli, Enterobacter* spp*, Citrobacter* spp, *Pseudomonas aeruginosa*, *Proteus* spp, *Streptococcus* spp, *Salmonella* spp*, Shigella* spp*, Campylobacter* spp [15, 16]. The World Health Organization (WHO) published the global Pathogen Priority List which contains selected bacteria pathogens for which new treatments are urgently needed. *Enterococcus faecium, Staphylococcus aureus, Klebsiella pneumoniae, Acinetobacter baumannii, Pseudomonas aeruginosa,* and *Enterobacter* spp (ESKAPE) are the pathogens, designated as “critical priority status” [17]. Out of these four are Gram-negative organisms.

The presence of resistant bacteria in hospital environments are a critical component of nosocomial infection. Hospital door handles contaminated with pathogenic bacteria and ESBL producing isolates increase the risk for infections and may be the key factor in epidemiology of ESBL-producing bacterial infection not only in a hospital setting but also in community. Door handles were pointed in this study because these are frequently touched surfaces shared by healthcare workers, patients and visitors but most neglected from cleaning or disinfection procedures. To our knowledge, there have been no published data available on the contamination of door handles of the hospitals by Gram negative pathogens and ESBL Producers with their antibiotic susceptibility pattern in Pokhara Metropolitan City, Nepal to date. Thus, this study was aimed to provide the baseline data on prevalence of ESBL producers and diversity and distribution of Gram-negative bacterial contamination along with their antibiotic resistance pattern from doorhandles in selected hospitals from Pokhara, Nepal. The isolation of pathogenic Gram-negative bacteria and ESBLs in hospital door handles and subsequent detection of high drug resistance patterns indicates a potentially serious public health challenge that strengthens the need for the effective and routine cleaning of door-handles in hospitals. The findings of this study might provide insight into the role of hospital-associated fomites, such as door handles, in the transmission of drug-resistant bacteria and the epidemiology of ESBL-producing bacterial infections.

## Methods

### Study site and design

The study was conducted in selected hospitals in Pokhara Metropolitan, City, Western Nepal. The Pokhara valley is located in central Nepal between 27˚55'-28˚23' north latitude and 83˚48'-84˚11' east longitude. A cross-sectional study was carried out following random sampling technique. The hospitals were selected randomly.

### Sample collection and processing

A total of 100 swab samples were collected from the door handles/knobs of two selected hospitals of Pokhara Metropolitan City, Nepal. The sterile cotton wool swabs were moistened with 5 ml of normal saline added to the swabs case and excesses were removed by pressing the swab stick against the inner side of the tube according to Chesebrough, 2000 [18]. Individual moistened sterile cotton swabs were used to swab the door handles/knobs.

The swab was wiped firmly on the entire surface of the door handles/knobs. It was then introduced into a test tube containing sterile Brain Heart Infusion (BHI) broth, labelled, kept below 4^0^C and transported to the laboratory of Janapriya Multiple Campus (JMC), Pokhara for analysis within two hours.

### Isolation and identification of bacteria

All the samples were processed by standard bacteriological procedures [18]. Collected samples were pre-enriched with BHI for 24 h, overnight incubation, at 37 °C to dislodge adhered bacteria. Each sample after pre-enrichment in sterile BHI broth, was aseptically sub cultured using streak plate method on MacConkey agar and incubated at 37 °C for 24 h [18]. The MacConkey agar plates were examined for cultural characteristics. Different colonies on the MacConkey agar plates were picked carefully and inoculated on nutrients agar plate to obtain pure growth. Bacteria identification was done using the pure culture on the Nutrient Agar plates. The identity of the isolates was confirmed by standard Laboratory methods which included colony morphology, Gram staining, biochemical test and other phenotypic characteristics [18].

### Antibiotics susceptibility test

Pure cultures of identified organisms were plated onto nutrient agar prior to all susceptibility tests. Antibiotic susceptibility testing was performed by the Kirby–Bauer disc diffusion method. The antibiotic disks were firmly placed on the sterile Mueller–Hinton agar plates (HI media, Mumbai, India) seeded with the tested strains, standardized to 0.5McFarland’s turbidity standard and incubated at 37 °C and interpreted according to the Clinical and Laboratory Institute Standard (CLSI) guidelines [19]. The discs of antibacterial agents used in this study contained amikacin (30 µg), norfloxacin (10 µg), imipenem (30 µg), cotrimoxazole (1.25/23.75 mcg), cefotaxime (30 µg), ceftazidime (30 µg), ciprofloxacin (5 µg), amox/clav (Amc30), tetracycline (30 µg), gentamicin (10 µg), nitrofurantoin (300 μg) and piperacillin (1.25/23.75 μg). *Escherichia coli* (ATCC 25922) and *Klebsiella pneumoniae* (ATCC 700603) were used as the quality control strains. Bacterial isolates that were resistant to minimum one agent in three or more than three antibiotic groups were categorized as multidrug-resistant (MDR) [20].

### Screening and confirmation of ESBL

Screening test for ESBL detection was done according to the CLSI guidelines [19]. Isolates showing inhibition zone size of ≤ 22 mm with ceftazidime (30 μg) and ≤ 27 mm with cefotaxime (30 μg) were interpreted as screening test positive for ESBL production [19].

The presumptive ESBL-positive isolates from screening methods were retested for ESBL production by the Double Disc Synergy Test according to the CLSI guidelines [19]. Briefly, set of two discs containing extended-spectrum cephalosporin [cefotaxime (30 μg) or ceftazidime (30 μg) alone and with a clavulanic acid combination (10 μg) were placed on-center spacing 25 mm apart on a Mueller Hinton Agar (HiMedia, India) plates inoculated with a bacterial suspension compared with 0.5 McFarland turbidity standard. Zone diameters were measured after overnight incubation at 37 °C. Bacterial isolates resistant to cefotaxime (zone diameter ≤ 27 mm) or ceftazidime (zone diameter ≤ 22 mm) and an increase of more than ≥ 5 mm in zone diameter with the discs containing clavulanic acid was confirmed to be ESBL-producers. Control strains of *Escherichia coli* (ATCC 25922) and *Klebsiella pneumoniae* (ATCC 700603) were used in parallel as a part of quality control.

### Data collection and analysis

Data entry and analysis were performed using GraphPad Prism software for window (version 6). A value of *p* ≤ 0.05 was assumed wherever applicable and 95% confidence intervals along with the exact *p*-values were presented. Data were presented in appropriate table, figures by using counts and calculating percentages, rate etc. Data were computed and described using counts and percentages. The ANOVA test was employed in comparing the distribution of MDR among ESBL producing Gram negative isolates at 95% confidence limit.

## Results

### Identification of isolated Gram-positive and Gram- negative bacteria

**Fig. 1:**
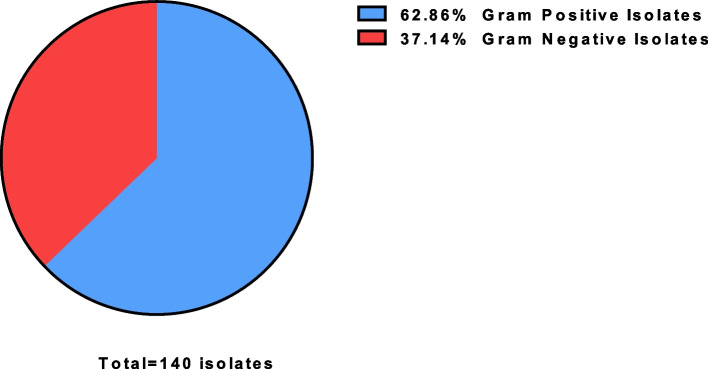
Percent number of Gram positive and Gram negative bacteria among the total isolates obtained from the door handles/knobs

A total of 140 isolates were found on the door handles obtained from 96 culture positive samples out of 100 samples processed. The Gram stain identified that (88/140) 62.86% of the bacteria found on the door handles were Gram positive and (52/140) 37.14% of the isolates were Gram negative (Fig. 1).

### Degree of growth of bacteria isolated from contaminated door handles/knobs

Table 1 shows the bacteria isolates and degree of growth from various door handles. The type of growth was indicated in this table as follows;

-  +  = one or few colonies,
-  +  +  = scanty growth,
-  +  +  +  = moderate growth and.
-  +  +  +  +  = profuse growth.

**Table 1:** Bacterial isolates and degree of growth from various door handles of different hospitals

**Sites**	**Bacterial isolates**	**Degree of growth**
**OPDs**	*Klebsiella pneumoniae*	(+ + + +)
	*Escherichia coli*	(+ + + +)
	*Staphylococcus aureus*	(+ + + +)
	Coagulase negative *Staphylococcus*	(+ + +)
	*Pseudomonas aeruginosa*	(+ + +)
	*Bacillus* spp	(+ + +)
	*Acenetobacter* spp	*(*+ + +)
	*Enterobacter* spp	(+ + +)
	*Diptheroids*	(+ +)
	*Micrococcus* spp	(+ +)
	*Proteus vulgaris*	(+ +)
	*Enterococcus* spp	( +)
	*Streptococcus pneumoniae*	( +)
**General wards**	*Staphylococcus aureus*	(+ + + +)
	*Escherichia coli*	(+ + + +)
	Citrobacter spp	(+ + + +)
	*Klebsiella pneumoniae*	(+ + + +)
	*Enterobacter* spp	(+ + + +)
	*Pseudomonas aeruginosa*	(+ + +)
	*Streptococcus pneumoniae*	(+ + +)
	*Proteus vulgaris*	(+ + +)
	*Bacillus* spp	(+ + +)
	*Acenetobacter* spp	(+ + +)
	Coagulase negative *Staphylococcus*	(+ +)
	*Enterobacter* spp	(+ +)
	*Enterococcus* spp	(+ +)
	*Micrococcus* spp	(+ +)
	*Diptheroids*	(+ +)
**Intensive care units**	*Escherichia coli*	(+ +)
	*Stapylococcus aureus*	(+ +)
	Coagulase negative *Staphylococcus*	(+ +)
	*Streptococcus pneumonia*	(+ +)
	*Pseudomonas aeruginosa*	(+ +)
	*Micrococcus* spp	(+ +)
	*Bacillus* spp	(+ +)
	*Diptheroids*	( +)
	*Klebsiella pneumoniae*	( +)
**Emergency**	*Klebsiella oxytoca*	(+ + + +)
	*Escherichia coli*	(+ + + +)
	*Staphylococcus aureus*	(+ + + +)
	*Citrobacter* spp	(+ + +)
	*Streptococcus pneumoniae*	(+ + +)
	Coagulase negative *Staphylococcus*	(+ + +)
	*Acenetobacter* spp	(+ + +)
	*Pseudomonas aeruginosa*	(+ +)
	*Bacillus* spp	(+ +)
	*Enterococcus* spp	(+ +)
	*Proteus mirabilis*	(+ +)
	*Diptheroids*	( +)
**Laboratory**	*E. coli*	(+ + + +)
	*Staphylococcus aureus*	(+ + + +)
	*Proteus vulgaris*	(+ + +)
	*Klebsiella pneumoniae*	(+ + +)
	*Klebsiella oxytoca*	(+ + +)
	*Enterobacter* spp	(+ + +)
	Coagulase negative *Staphylococcus*	(+ + +)
	*Micrococcus* spp	(+ + +)
	*Bacillus* spp	(+ + +)
	*Citrobacter* spp	(+ +)
	*Pseudomonas aeruginosa*	(+ +)
	*Diptheroids*	(+ +)
	*Streptococcus pneumoniae*	(+ +)
**Staff Room**	*Staphylococcus aureus*	(+ +)
	*Proteus mirabilis*	(+ +)
	*Escherichia coli*	(+ +)
	*Bacillus* spp	(+ +)
	Coagulase negative *Staphylococcus*	(+ +)
	*Acenetobacter* spp	(+ +)
	*Diptheroides*	(+ +)
	*Micrococcus* spp	(+ +)
	*Streptococcus pneumoniae*	( +)
**Radiology Room**	*Staphylococcus aureus*	(+ +)
	Coagulase negative *Staphylococcus*	(+ +)
	*Enterobacter* spp	(+ +)
	*Bacillus* spp	(+ +)
	*Pseudomonas aeruginosa*	(+ +)
	*Enterococcus* spp	(+ +)
	*Diptheroids*	(+ +)
	*Micrococcus* spp	(+ +)
**Operation Theatre**	*Staphylococcus aureus*	(+ +)
	Coagulase negative *Staphylococcus*	( +)
	*Bacillus* spp	( +)
	*Enterobacter* spp	( +)
	*Pseudomonas aeruginosa*	( +)
	*Micrococcus* spp	( +)
	*Diptheroids*	( +)
**Toilets**	*Staphylococcus aureus*	(+ + + +)
	*Bacillus* spp	(+ + + +)
	*Escherichia coli*	(+ + + +)
	*Klebsiella oxytoca*	(+ + + +)
	*Acenetobacter* spp	(+ + +)
	*Pseudomonas aeruginosa*	(+ + +)
	Coagulase negative *Staphylococcus*	(+ + +)
	*Proteus mirabilis*	(+ + +)
	*Enterobacter* spp	(+ + +)
	*Micrococcus* spp	(+ +)
	*Diptheroids*	(+ +)

KEYS: +  = One or few colonies +  +  = Scanty Growth +  +  +  = Moderate Growth +  +  +  +  = Profuse Growth

### Distribution pattern of Gram-negative bacteria isolated from various door handles/knobs

The most common Gram-negative organism isolated in this study was *Escherichia coli* 15(28.85%), followed by *Klebsiella spp* 11(21.15%), *Pseudomonas aeruginosa* 8 (15.38%), *Proteus spp* 6 (11.54%), *Enterobacter spp* 5 (9.62%), *Acenetobacter spp* 4 (7.69%) and *Citrobacter spp* 3 (5.77%) (Table 2).

**Table 2:** Percentage distribution pattern of Gram-negative bacteria isolated from various door handles/knobs

**Organism Identified**	**Number**	**Frequency**
*Escherichia coli*	15	28.85%
*Klebsiella* spp	11	21.15%
*Pseudomonas aeruginosa*	8	15.38%
*Proteus* spp	6	11.54%
*Enterobacter* spp	5	9.62%
*Acenetobacter* spp	4	7.69%
*Citrobacter* spp	3	5.77%
**Total**	**52**	**100%**

### Antibiotic susceptibility test of the isolated Gram-negative bacteria

Most of the Gram-negative bacilli isolated were found to be 100% resistance to Cotrimoxazole (COT), Gentamycin (GEN) and Amoxicillin + Clavulanate (AMC). The most effective drug of choice were Amikacin (AK), Nitrofurantoin (NIT), Norfloxacin (NOR), Ciprofloxacin (CIP), Tetracycline (TE) (80%) and Imipenem (IPM) for many isolates. Various antibiotics were used for antibiotic susceptibility pattern determination using Kirby Bauer disc diffusion method. *E. coli* was found to be 100% resistant to Amoxicillin + Clavulanate, Cotrimoxazole and Gentamycin. The most effective drug of choice were Amikacin and Nitrofurantoin showing 100% sensitivity followed by Ciprofloxacin (86.6%), Tetracycline (80%), Norfloxacin (80%), Imipenem (66.6%), Piperacillin (46.6%), Cefotaxime (26.6%) and Ceftazidime (26.6%) (Table 3).

**Table 3:** Antibiotic susceptibility pattern of the isolated Gram-negative bacteria

**Pathogens**	**Total Number Of Isolates**	**Number (%) of isolates Sensitive to**											
		**CTX**	**CEFTAZIDIME**	**AMC**	**GEN**	COT	CIP	TE	**NORKor**	**IPM**	**AK**	**NIT**	PI
*Escherichia coli*	15	4(26.6)	4(26.6)	0	0	0	13(86.6)	12(80)	12(80)	10(66.6)	15(100)	15(100)	7(46.6)
*Klebsiella spp*	11	2(18.1)	2(18.1)	0	0	0	7(63.6)	9 (81.8)	11(100)	10(90.9)	11(100)	7(63.6)	0
*Pseudomonas aeruginosa*	8	0	0	0	0	0	0	0	0	0	2(25)	1(12.5)	0
*Proteus spp*	*6*	2(33.3)	2(333)	1(16.6)	0	0	4(66 6)	2(33.3)	4(66 6)	4(66 6)	5(83.3)	6(100)	0
*Enterobacter spp*	*5*	2(40)	2(40)	3(60)	0	0	1(20)	1(20)	5(100)	3(60)	5(100)	5(100)	5(100)
*Acenetobacter spp*	4	0	0	0	0	0	0	0	2(50)	0	1(25)	1(25)	0
*Citrobacter spp*	3	1(33.3)	2(66.6)	1(33 3)	1 (33.3)	0	1(333)	1(33.3)	3(100)	2(66.6)	3(100)	3(100)	2(66.6)

Antibiotic susceptibility pattern in *Klebsiella* species showed different than that of *E. coli* isolates. *Klebsiella* species showed 100% resistance to Amoxicillin + Clavulanate, Cotrimoxazole and Piperacillin (PI). The most effective drug of choice was Norfloxacin and Amikacin showing 100% sensitivity followed by Imipenem (90.9%), Tetracycline (81.8%), Nitrofurantoin (63.6%), Ciprofloxacin (63.6%), Cefotaxime (CTX) (18.1%) and Ceftazidime (18.1%) (Table 3).

However, *Pseudomonas aeruginosa* showed 100% resistance to almost all antibiotics tested except sensitive to Amikacin (25%) and Nitrofurantoin (12.5%). Similarly, *Acenetobacter spp* also showed 100% resistance to most antibiotics tested and sensitive to only Norfloxacin (50%), Amikacin (25%) and Nitrofurantoin (25%) (Table 3).

*Proteus* species showed 100% resistance to Gentamycin, Cotrimoxazole and Piperacillin. The most effective antibiotic was Nitrofurantoin with 100% sensitivity followed by Amikacin (83.3), Imipenem (66.6%), Norfloxacin (66.6%), Ciprofloxacin (66.6%), Tetracycline (33.3%), Cefotaxime (33.3%), Ceftazidime (33.3%) and Amoxicillin + Clavulanate (16.6%) (Table 3).

*Enterobacter* species showed100% resistance. to Gentamycin and Cotrimoxazole. The most effective drug of choice was Amikacin, Nitrofurantoin, Piperacillin and Norfloxacin showing 100% sensitivity followed by Amoxicillin + Clavulanate (60%), Cefotaxime (40%), Ceftazidime (40%) and Imipenem (40%) (Table 3).

*Citrobacter* species showed 100% resistant Cotrimoxazole. The most effective antibiotics were Amikacin, Nitrofurantoin and Norfloxacin (100%) followed by Imipenem (66.6%), Piperacillin (66.6%), Ceftazidime (66.6%), Cefotaxime (33.3%), Ciprofloxacin (33.3%), Gentamycin (33.3%), Amoxicillin + Clavulanate (33.3%) and Tetracycline (33.3%) (Table 3).

### Prevalence of ESBLs among the isolated Gram-negative bacteria

**Fig. 2:**
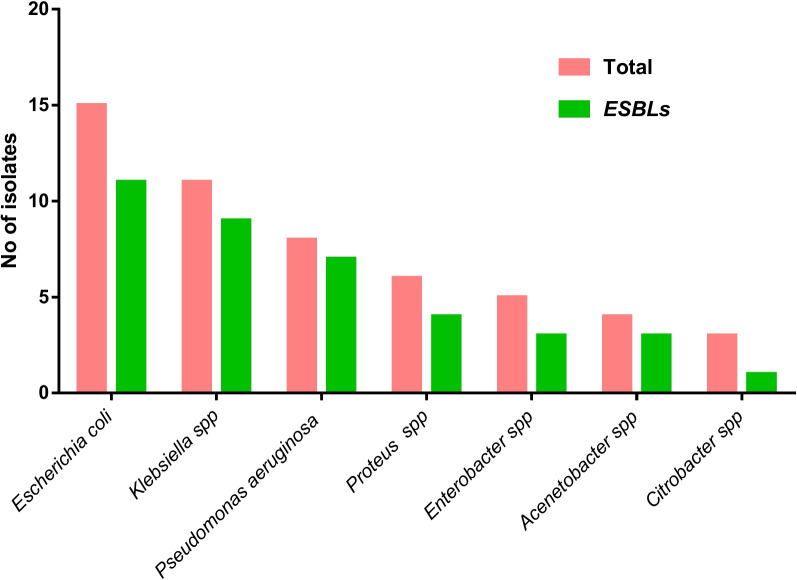
Distribution of ESBL producing Gram negative isolates

Out of total 140 isolates 38 were found to be ESBLs. The overall prevalence of ESBLs in this study was 27.14% (38/140). Total of 52 Gram negative bacilli were observed. Out of total 15 *Escherichia coli* isolated, 11(73.3%); out of total 11 *Klebsiella* spp 9 (81.8%); out of total 8 *Pseudomonas* spp isolated 7 (87.5%); out of 6 *Proteus* spp isolated 4 (66.6%); out of total 5 *Enterobacter* spp 3 (60%), out of total 4 *Acenetobacter* spp 3 (75%) and out of 3 *Citrobacter* spp 1 (33.3%) were found to be Extended B-Lactamase Producers (ESBLs) (Fig. 2).

### Distribution of Multi drug Resistance (MDR) among ESBL producing isolates

**Fig. 3:**
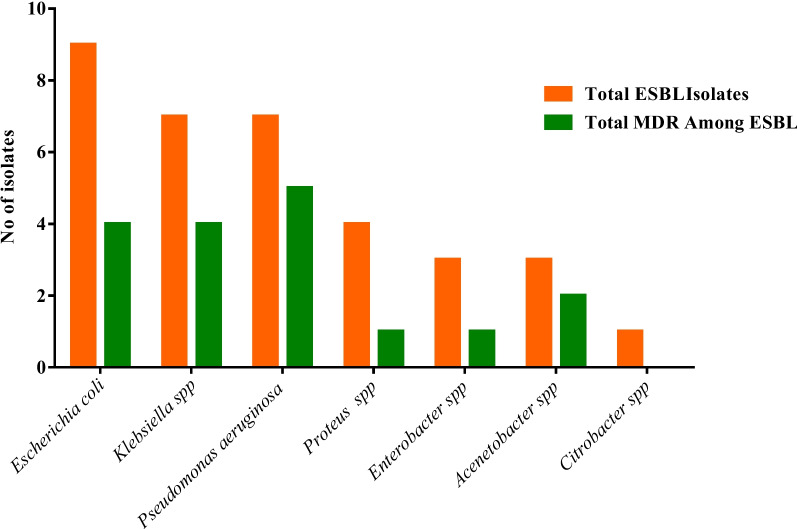
Distribution of MDR among ESBL producing Gram negative isolates

Out of 38 ESBL isolate, 16(42.11%) were Multi Drug Resistant (MDR) and 22(57.89%) were Non MDR. Out of total MDR isolate, 4 (10.53%) were *E. coli* (25%), 4(25%) *Klebsiella spp*, 5(31.25%) *Pseudomonas* spp, 1(6.25%) *Proteus* spp, 1(6.25%) *Enterobacter* spp, 2(12.5%) *Acenetobacter* spp. Statistically there was no significant association between the ESBL producing isolates among MDR phenomena (*p* > 0.05) (Fig. 3).

## Discussions

The presence of drug resistant bacteria in hospital environments are a critical component of nosocomial infection. Contaminated intermediate objects represent a common transition of transmission between patients, from visitors to patients or from healthcare workers to patients [21, 22], which also impact on the choice of antibiotic prophylaxis for surgeries [23]. We recognize some flaws in this study due to a lack of standard or widely accepted method for collecting and isolating bacterial isolates from surfaces, which can make it difficult to compare results from different studies and to accurately determine the bacterial composition of a surface. In addition, Many bacterial species are able to enter a dormant or resistant state when exposed to adverse conditions, such as low moisture or low nutrient levels. These bacteria may not be detected by traditional culture methods, making it difficult to isolate and study them. However, in this study, the enrichment culture, culture on selective medium were performed to isolate the bacteria along with necessary staining and biochemical tests for the phenotypic characterization of the isolated contaminants and pathogens using the standard microbiological techniques and all the antimicrobial tests were performed only after subculturing the pure culture of the microorganisms after proper identification using standard Clinical Laboratories Standard Institute (CLSI) guidelines [19]. The double disk diffusion test performed in this study was only able to detect resistance to a limited number of antibiotics, and it did not provide information about the mechanisms of resistance or the susceptibility of bacteria to other antibiotics. In this regard, it is recommended to perform Whole Genome Sequencing to access the clonal distribution, resistance mechanism diversity and other molecular aspects of gram-negative identified bacterial species.

In this study, the level of contamination was higher in door handles of Outpatient Departments (OPDs), Emergency, Laboratory, General wards and Toilets, in that order as compared to Radiology Room, Staff rooms, Intensive Care Unit (ICU) and Operation Theatre (OT) which were lower. The lower level of contamination in ICU and OT could be attributed to the fact that they are not being used as frequently as other places studied, this is in agreement with the findings of Boone and Gerba (2010) [24] and Nworie et al.(2012) [15] who reported that the levels of contamination vary depending on the traffic, exposure and environment. Similarly, this is in agreement with the findings of Hedieh et al. (2012) [25], who found a significant correlation between the frequency of movement through a door and the degree to which it was contaminated.

This study also highlighted the presence of potential pathogenic Gram-negative bacteria in door handles of hospitals. *Escherichia coli*, *Klebsiella* spp, *Pseudomonas* spp, *Acenetobacter* spp, *Enterobacter* spp, *Proteus* spp and *Citrobacter* spp were the main Gram-negative bacteria isolated in this research work so far.

The fact that bacteria of the Enterobacteriaceae found on different door handles may indicate faecal contamination of the hands as the origin. This might be due to the fact that most people may fail to wash their hands and contaminate with faecal and urinal material due to lack of the concept of hand hygiene to stop the spread of infectious agents, which was corresponded with the work of Zhad et al. (1998) [26], who reported that human hands served as the vehicle of transmission of these pathogenic microrganisms. Similarly, Orskov et al. (1997) reported the Enteric pathogens that may be present on the hand include *Escherichia coli*, *Salmonella typhi*, *Shigella* spp., *Clostridium perfringes*, *Giardia lamblia*, Norwalk virus and Hepatitis A virus; *Pseudomonas aeruginosa*, *S. aureus*, *Proteus mirabilis*, *Citrobacter freundii*, *Enterobacter* spp; *Streptococcus* spp, *Klebsiella* spp [27]. The isolation of pathogenic bacteria from fomites indicates that they can be vehicles for pathogens transfer. Gram negative sepsis, urinary tract infections are most commonly caused by *E. coli* and *Klebsiella* spp. The presence of these pathogenic bacteria on environmental surfaces such as door handles poses a potential risk to vulnerable, immune-compromised individuals.

Most of the Gram-negative bacilli isolated were found to be 100% resistance to Amoxicillin + Clavulanate, Cotrimoxazole and Gentamycin. The most effective drug of choice were Amikacin, Nitrofurantoin, Norfloxacin, Ciprofloxacin, Tetracycline (80%) and Imipenem for many isolates. However, *Pseudomonas aeruginosa* showed 100% resistance to almost all antibiotics tested except sensitive to Amikacin (25%) and Nitrofurantoin (12.5%). Similarly, *Acenetobacter spp* also showed 100% resistance to most antibiotics tested but was sensitive to only Norfloxacin (50%), Amikacin (25%) and Nitrofurantoin (25%). This is in agreement with the findings of other studies Beta-lactam antimicrobial agents exhibit the most common treatment for bacterial infections and continue to be the prominent cause of resistance to beta-lactam antibiotics among Gram negative bacteria worldwide. The persistent exposure of bacterial strains to a multitude of beta-lactams has induced dynamic and continuous production and mutation of b-lactamases in these bacteria, expanding their activity even against the newly developed b-lactam antibiotics. These enzymes are known as extended-spectrum beta-lactamases (ESBLs) [28, 29]. Treatment of these multiple drug resistant organisms is a deep scientific concern. Production of extended-spectrum beta-lactamases is a significant resistance-mechanism that impedes the antimicrobial treatment of infections caused by *Enterobacteriaceae* and is a serious threat to the currently available antibiotic Armory [28, 29].

Out of total 15 *Escherichia coli* isolated, 11(73.3%); out of total 11 *Klebsiella spp* 9(81.8%); out of total 8 *Pseudomonas spp* isolated 7(87.5%); out of 6 *Proteus spp* isolated 4(66.6%); out of total 5 *Enterobacter spp* 3(60%), out of total 4 *Acenetobacter spp* 3(75%) and out of 3 *Citrobacter spp* 1 (33.3%) were found to be Extended B-Lactamase Producers (ESBLs). Overall prevalence of ESBLs in this study was 27.14% (38/140). Similar prevalence rates of ESBL in urinary isolates in Nepal were reported by the findings of Manandhar et al. (2006) [30].

In this study most of the Gram-negative isolates were Multidrug resistance and resistant to Cefotaxime, Ceftazidime, Amoxicillin + clavulanate, Gentamycin, and Cotrimoxazole which is in agreement with other study who also found 100% resistant to Cefotaxime and Ceftazidime [31]. For ESBL producing *E coli* and *Klebsiella* species, the Amikacin, Norfloxacin, Nitrofurantoin, Tetracycline and Imipenem were found to be effective drugs of choice likewise in the study done by Stoesser et al. (2015) [31] reported 96% isolates susceptible to Nitrofurantoin. The increasing use of broad spectrum cephalosporins has become one of the major factors responsible for the high rate of ESBL producing microorganisms [32]. Indiscriminate use of antibiotics and delay in seeking medical treatment could be other reason for high rate of resistance could be higher rates of resistance to various antimicrobials in different hospitals of Western region of Nepal.

Door handles are frequently touched surfaces shared by healthcare workers, patients and visitors but most neglected from cleaning or disinfection procedures. The rising antibiotic resistance to bacteria that might contaminate the door handles of hospitals is a major worry worldwide, but particularly in emerging and underdeveloped nations like Nepal; and there is lack of information regarding antimicrobial resistance patterns of commonly isolated pathogens, particularly in the study area. Hence, this study assessed the base line data on prevalence of ESBLs producers and diversity and distribution of Gram-negative bacterial contamination along with their antibiotic resistance pattern from doorhandles in selected hospitals from Pokhara, Nepal. The isolation of pathogenic Gram-negative bacteria and ESBLs in hospital door handles and subsequent detection of high drug resistance patterns indicates a potentially serious public health challenge that strengthens the need for the effective and routine cleaning of door-handles in hospitals.

Finally, a comprehensive survey and research on antibiotic resistance are required to analyze this disastrous national situation and develop management solutions.

Door handles are a well-documented breeding ground for pathogens and present a high risk common contact surface facilitating the transmission of potentially pathogenic microorganisms. Door handles design and material of handle itself contributes to the growth of bacteria and might play key role to control microbial transmission [33]. Novel door handles could be developed which might prove to be more resistant to microbial contamination than existing designs. This study revealed the presence of potentially pathogenic, multi drug resistant and ESBLs on door handles of the hospitals which increase the risk for nosocomial infections and may be the key factor in epidemiology of ESBL-producing bacterial infection not only in a hospital setting but also in community. Thus, monitoring and evaluation of hospital door handles should always be a vital procedure for infection control teams of hospitals to protect staff, visitors and vulnerable patients. Frequent hand washing and Door handle sanitisers or spray disinfectants should be recognised as an essential constituent in the fight against Hospital Acquired Infections. Heavy metal such as silver or copper could be used to reduce the microbial load of the door handles [33] because material of the handle itself contributes to the growth of bacteria.

## Conclusion

Isolation of pathogenic drug resistant Gram-negative bacteria and ESBLs from door handles of the hospitals is worrisome. Presence of such drug resistant bacteria including ESBLs in hospital door handles increase the risk for infection and may be the key factor in epidemiology of ESBL producing bacterial infection not only in a hospital setting but also in community. Therefore, hospital staffs, inpatients, outpatients and visitors should adopt the habit of hand washing practice after using the door handles in hospitals. Indeed, regular surveillance and routine surface disinfection of the hospital door handles should be done to prevent cross contamination.

## Data Availability

All data generated during this study are included in this article.

## Abbreviations

ESBL: Extended Spectrum β-Lactamase
WHO: World Health Organization
BHI: Brain Heart Infusion
CLSI: Clinical and Laboratory Institute Standard
MDR: Multi drug Resistance
OPDs: Outpatient Departments (OPDs)
ICU: Intensive Care Unit (ICU)
OT: Operation Theatre (OT)
